# Prevalence of Overweight and Obesity among Female Adolescents in Jordan: A comparison between Two International Reference Standards

**DOI:** 10.12669/pjms.292.3222

**Published:** 2013-04

**Authors:** Abdulrahman O. Musaiger, Mariam Al-Mannai, Reema Tayyem

**Affiliations:** 1 Abdulrahman O. Musaiger, Nutrition and Health Studies Unit, Deanship of Scientific Research, University of Bahrain and Arab Center for Nutrition, Bahrain.; 2 Mariam Al-Mannai, Department of Mathematics, College of Science, University of Bahrain, Bahrain.; 3 Reema Tayyem, Department of Clinical Nutrition and Dietetics, Faculty of Allied Health Science, The Hashemite University, Zarqa, Jordan.

**Keywords:** Adolescents, Overweight, Obesity

## Abstract

***Objective:*** To find out the prevalence of overweight and obesity among female adolescents in Jordan.

***Methodology:*** A cross-sectional survey on females aged 15–18 in Amman, Jordan, was carried out using a multistage stratified random sampling method. The total sample size was 475 girls. Weight and height were measured and body mass index for age was used to determine overweight and obesity using the IOTF and WHO international standards.

***Results:*** The prevalence of overweight and obesity decreased with age. The highest prevalence of overweight and obesity was reported at age 15 (24.4% and 8.9%, respectively). The WHO standard showed a higher prevalence of obesity than the IOTF standard in all age groups.

***Conclusions:*** Overweight and obesity are serious public health problems among adolescents in Jordan, using both international standards. A program to combat obesity among schoolchildren, therefore, should be given a high priority in school health policy in Jordan.

## INTRODUCTION

The nutrition and economic transitions which have happened in Jordan during the recent decades have influenced the nutrition and health situation. Chronic non-communicable diseases have become the main health problems, while the prevalence of infectious diseases has declined steeply.^1^ Consequently, obesity has risen as a major challenge to the health authority in Jordan. Studies have shown that obesity among both children and adults has reached an alarming level in Jordan.^2^^,^^3^ They documented that obesity during childhood is associated with impaired health-related quality of life.^4^ The present study therefore, aimed to explore the prevalence of obesity among adolescent girls in Amman, the capital of Jordan.

## METHODOLOGY

A school-based cross-sectional survey was carried out among girls aged 15–18 in Amman, Jordan. A multistage stratified random sampling method was used to select the girls. Amman was divided into four geographical areas, and then the secondary schools were selected proportionally to the student population in each area. Classes were selected by a simple random method for each secondary level (levels 10, 11 and 12). Only government schools were included in the study. The total sample obtained was 475 girls.

Weight and height were measured by trained nutritionists, with minimum clothes and without shoes. For the sake of comparison, overweight and obesity were calculated using two reference standards: the International Obesity Task Force (IOTF)^5^ and the World Health Organization (WHO) growth chart for children.^6^ The girls were divided into non-obese, overweight and obese.

## RESULTS

The prevalence of overweight and obesity among Jordanian adolescent girls is presented in Table-I. The proportion of overweight and obesity decreased as the age of the girls increased. The highest prevalence of overweight (24.4%) and obesity (8.9%) was reported at age 15. The WHO reference standard provided a higher prevalence of obesity than the IOTF standard. As for overweight, the WHO standard showed a lower prevalence at ages 15 and 18, but a comparable prevalence at ages 16 and 17.

**Table-I:** Prevalence of overweight and obesity among adolescent girls in Amman, Jordan, based on IOTF and WHO reference standards

*Age (years)*	*Sample size*	*Reference*	*Non-obese* *(%)*	*Overweight* *(%)*	*Obese* *(%)*
15	45	IOTF	66.7	24.4	8.9
		WHO	66.7	20.0	13.3
16	191	IOTF	74.9	19.4	5.7
		WHO	73.8	19.9	6.3
17	176	IOTF	81.3	14.8	3.9
		WHO	78.4	15.9	5.7
18	63	IOTF	85.7	14.3	0.0
		WHO	85.7	12.7	1.6

## DISCUSSION

In general, this study showed that the problem of obesity is of great concern among adolescent girls in Jordan, and there is a variation in the prevalence of overweight and obesity between the WHO and IOTF standards. Most of the studies on obesity among adolescents in Jordan were carried out in the northern area. The findings of the current study were similar to those reported in the northern area of Jordan for girls aged 13-18^4^ and for girls aged 14-17.^7^ When comparing our results for girls aged 15-16 with those reported in Amman^8^, our data showed a relatively higher proportion of overweight (19.9% vs. 17.5%), but a lower proportion of obesity (7.6% vs. 9.6%), using the IOTF standard.

Some factors that may be associated with the rising prevalence of obesity among children and adolescents in Jordan have been investigated. Khader et al^2^ found that watching television for more than two hours a day and a family history of obesity were significantly associated with increased risk of obesity among 6–12-year-old Jordanian children. Al-Kloub et al^8^ reported that the main predictors of obesity among 15–18-year-old Jordanian students were father’s education, high family income, working mother, family history and unhealthy diet. Physical inactivity, mother’s education and number of family members were found to be negatively associated with obesity among Jordanian adolescents aged 14–17; however, eating breakfast regularly, high intake of fried food and perceived stress level were found to be positively associated with obesity.^7^ It is widely believed that many socio-cultural and lifestyle factors are also responsible for the high proportion of obesity among schoolchildren in Jordan.^7^

This study showed for the first time a comparison between two international standards to determine overweight and obesity among Jordanian adolescents. A selection of reference standards, therefore, should be considered when interpreting the prevalence of obesity among schoolchildren. The findings of this study raise the need for an intervention school program to prevent and control obesity in schoolchildren in Jordan.
